# Human papillomavirus-mediated carcinogenesis and HPV-associated oral and oropharyngeal squamous cell carcinoma. Part 2: Human papillomavirus associated oral and oropharyngeal squamous cell carcinoma

**DOI:** 10.1186/1746-160X-6-15

**Published:** 2010-07-15

**Authors:** Liviu Feller, Neil H Wood, Razia AG Khammissa, Johan Lemmer

**Affiliations:** 1 Department of Periodontology and Oral Medicine, University of Limpopo, Medunsa Campus, South Africa

## Abstract

Human papillomavirus (HPV) infection of the mouth and oropharynx can be acquired by a variety of sexual and social forms of transmission. HPV-16 genotype is present in many oral and oropharyngeal squamous cell carcinomata. It has an essential aetiologic role in the development of oropharyngeal squamous cell carcinoma in a subset of subjects who are typically younger, are more engaged with high-risk sexual behaviour, have higher HPV-16 serum antibody titer, use less tobacco and have better survival rates than in subjects with HPV-cytonegative oropharyngeal squamous cell carcinoma. In this subset of subjects the HPV-cytopositive carcinomatous cells have a distinct molecular profile.

In contrast to HPV-cytopositive oropharyngeal squamous cell carcinoma, the causal association between HPV-16 and other high-risk HPV genotypes and squamous cell carcinoma of the oral mucosa is weak, and the nature of the association is unclear.

It is likely that routine administration of HPV vaccination against high-risk HPV genotypes before the start of sexual activity will bring about a reduction in the incidence of HPV-mediated oral and oropharyngeal squamous cell carcinoma.

This article focuses on aspects of HPV infection of the mouth and the oropharynx with emphasis on the link between HPV and squamous cell carcinoma, and on the limitations of the available diagnostic tests in identifying a cause-and-effect relationship of HPV with squamous cell carcinoma of the mouth and oropharynx.

## Introduction

Human papillomaviruses have been categorized by their genotypes into low-risk and high-risk types according to the risk of that virus causing squamous cell carcinoma of the uterine cervix [1]. Infection of the uterine cervix with any human papillomavirus (HPV) genotype is associated with high-risk sexual behaviour, particularly if started at a younger age; and persistent infection of the uterine cervix with high-risk HPV genotypes, especially HPV-16 and HPV-18, is essential for the development of squamous cell carcinoma (SCC) [1-3]. Recent evidence also incriminates high-risk HPV-genotypes in the pathogenesis of oral and oropharyngeal SCC [4-21], and it will be the purpose of this paper to explore this relationship.

HPV infection of the mouth and of the oropharynx, like HPV infection of the uterine cervix, is associated with high-risk sexual behaviour, in particular with orogenital sex; and high-risk HPV genotypes, in particular HPV-16, are present in many oral and oropharyngeal SCC where in some cases they probably play an essential aetiological role [17]. Persons with oropharyngeal SCC in which HPV can be detected intracellularly have a better prognosis than persons with HPV-cytonegative oropharyngeal SCC [11,14].

## The circumstantial evidence for a link between HPV and squamous cell carcinoma of the mouth and oropharynx

In order to prove a causal relationship between HPV and SCC of the mouth and oropharynx, as has been proven in the case of SCC of the cervix uteri, there should be evidence that in a significant number of cases of apparently normal oral or oropharyngeal epithelium infected with HPV, in time SCC will develop. The demonstration of HPV DNA, even of high-risk HPV oncogenes in squamous cell carcinoma is not in itself sufficient evidence of oncogenesis by the HPV in that context. HPV may well have been either present but a non-participant during the oncogenesis, or have been superimposed upon the malignancy.

On the other hand, absence of HPV DNA from any carcinoma does not exclude the theoretical possibility of its having played some role in the initiation of the malignancy since HPV infections are frequently transient [7]. In such a 'hit and run' situation, HPV may incite initial transformation in cells that subsequently lose their HPV DNA sequences during carcinogenesis [8]. However, this is highly improbable since persistence of oncoproteins E6, E7 of the high-risk HPV genotypes appears to be necessary for the perpetuation of HPV-associated malignancy, as is evident from the presence of HPV DNA in the cells of SSC of the uterine cervix [9].

The local viral load and viral distribution, the clonality of HPV infection, the mechanisms of HPV oncogene transcription, and the specific site of viral integration are all factors critical to the understanding of HPV oncogenesis; and the testing for these factors is as complex and as multifaceted as the complexity of the process itself.

*In situ *hybridization assays for HPV DNA can provide data on the presence of HPV in different cells, but have limited sensitivity for certain HPV genotypes and cannot demonstrate oncogene transcription. Viral oncogene expression can be demonstrated by the polymerase chain reaction (PCR) technique, but this does not provide information about the viral load and the distribution of HPV DNA [9]. As PCR can detect very small fragments of HPV DNA that may just be tissue contamination or biologically insignificant HPV infection, PCR findings without quantifying the DNA viral load or identifying HPV transcriptional activity are not significant in relation to HPV oncogenesis [22,23]. Neither PCR nor *in situ *hybridization tests can pinpoint the specific site of viral integration in the genome [9]. PCR combined with *in situ *hybridization can detect HPV-infected cells with low viral loads, and can also elucidate the distribution of HPV DNA within the tumour [10].

Circumstantial evidence for the role of high-risk HPV types in the pathogenesis of SCC of the mouth and oropharynx can be found, firstly, in the presence of high-risk HPV genomic sequences and expression of transcriptionally active E6/E7 oncoproteins in the malignant cell nuclei of the tumour and of its metastases; secondly, in HPV DNA integration in the cellular genome; and thirdly, in the existence of substantial viral DNA copy-numbers [9,11,12,24].

In relation to HPV viral load, although there is a clearly demonstrated association between increased HPV DNA copy-number (viral load) and increased risk of cervical cancer, this viral load is not a reliable predictor of HPV-induced progression to cervical cancer; and presumably, viral load will be no more reliable as a predictor of HPV-induced progression to oral and oropharyngeal cancer. Determination of viral load cannot discriminate between HPV infection of a few cells with a large number of HPV DNA copies each, and of many cells with a few DNA copies each; or between recent HPV infection and long-standing infection [25].

Regarding HPV DNA integration into the cellular genome, although this molecular event is a strong indication of the oncogenic role of the virus, the presence of high HPV DNA copy-numbers and transcriptionally active (high risk) E6/E7 mRNA in HPV cytopositive SCC of the oropharynx is not necessarily dependant on viral integration and can occur when the virus is in an episomal form [26].

## Acquisition of oral and oropharyngeal HPV infection

Both oral and oropharyngeal HPV infection and oral and oropharyngeal SCC are associated with the practice of orogenital sex and with the high-risk sexual behaviour of cohabiting with many partners, particularly when started at a younger age [7,12,15,17,19,27]. In a study primarily aimed at vulvogenital HPV infection, tobacco smoking and increasing age were found to be risk factors associated with increased frequency of persistent oral HPV infections in women [28]. This appears to be because tobacco-mediated and age-related local genetic and immune dysregulation renders the tissues more susceptible to HPV infection [28].

Although oral and oropharyngeal HPV infections are primarily sexually acquired, mouth to mouth contact between partners and between family members, autoinoculation, and vertical birth-transmission are also routes whereby HPV infection of oral and oropharyngeal sites can be established [15,27,29].

As oral and oropharyngeal subclinical HPV infection is not uncommon, it is possible that the epithelium may serve as a reservoir of the virus, and when activated the virus may play a role in HPV-associated oral and oropharyngeal SCC.

## The role of HPV in oral and oropharyngeal SCC

In epidemiological studies, SCC of the head and neck is frequently treated as a homogeneous group, and the various component carcinomata (oral, oropharyngeal, laryngeal, nasopharyngeal, hypopharyngeal etc.) are not often separated out statistically. The reported rates of detection of HPV DNA in head and neck SCC range from 0 to 100% [15,30]. This extreme variation in reported prevalence may be owing to lumping together of essentially different lesions; to small sample numbers; and to differences in the sampling techniques; in the ethno-geographic origins of the subjects examined; and in the HPV detection methods applied [13,23,31].

Understanding of the role of HPV in the pathogenesis of oral and oropharyngeal SCC is further clouded by inconsistencies in the evidence brought about by differences in methods of tissue collection and preservation; by the use of molecular assays and HPV DNA probes with different specificities and sensitivities; by low viral load in these carcinomata; by lack of adequate controls; and by the inability to identify and assess the influences of other confounding factors [10,16,23]. However, it is generally accepted that HPV DNA is detected in about 26% of biopsy specimens of SCC of the head and neck [6,15]; and that these neoplasms, in particular SCC of the tonsil, contain HPV DNA more frequently than any other SCC of the head and neck [6,11,23,32]. In a meta-analysis of data from 94 studies of a total of 4580 specimens, Miller and Johnston (2001) determined that the prevalence of HPV in normal oral mucosa and in oral SCC is likely to be 10% and 46.5%, respectively [16].

Coinfection with HPV-16 together with one or more other HPV types is not uncommon [10,18]. HPV-16 DNA was found to be the most prevalent HPV genotype in HPV-cytopositive oral and oropharyngeal SCC [6,15,18] and was detected in about 75% of cases of HPV-cytopositive oral SCC and in about 90% of cases of HPV-cytopositive oropharyngeal SCC [17-19]. A recent meta-analysis of data from 17 studies determined that there is a significant causal association between HPV-16 and oropharyngeal SCC, but only a weak association in the case of oral SCC [23].

Serum antibodies against L1, E6 and E7 proteins of HPV-16 were detected in well over 60% of persons with oropharyngeal SCC [17]. Since antibodies to HPV-16 capsid protein L1 are strongly associated with oral and oropharyngeal SCC, and since these antibodies are evidence of long-term exposure to HPV-16, it is possible, indeed probable, that exposure to HPV-16 precedes the development of oropharyngeal SCC by several years [7,15,17]. However, this observation must be interpreted with caution since other HPV infections, for instance anogenital and oral warts will increase HPV antibody titres, and this can confound the observed association between serum HPV antibody levels and oral and oropharyngeal SCC [7]. As is the case with the virus itself, HPV-16 seropositivity is strongly associated with increased risk of developing HPV-cytopositive oropharyngeal SCC, but there is only a weak association for oral SCC [32,33].

Owing to the non-specificity of clinical sampling methods for HPV and to the confounding effect of benign HPV infection in the mouth or elsewhere, prediction of development of HPV-associated oral and oropharyngeal SCC can not yet be made [12,15,34].

HPV-associated and non HPV-associated (tobacco/alcohol related, idiopathic) oral and oropharyngeal SCC are different in cytogenetic profiles, clinical characteristics and courses of the disease [11,12]. While HPV-associated cytopositive oral and oropharyngeal SCC is thought to be initiated and maintained by high-risk HPV E6/E7 oncoprotein-induced dysregulation of cell cycle control mechanisms, leading to genomic instability [12,17], HPV-cytonegative oral and oropharyngeal SCC often show mutation of p53 tumour-suppressor gene, frequent loss of heterozygosity (LoH) at chromosomal loci 3p, 9p and 17p, normal or increased levels of pRb, and decreased levels of p16^INK4A ^[35,36]. HPV-associated and non-HPV-associated pathogenic mechanisms result in distinctly different cellular molecular characteristics [12,20].

It is not yet clear whether the use of tobacco/alcohol and HPV are, or are not synergistic in the aetiopathogenesis of oral and oropharyngeal SCC [11,12], but in a recent case-controlled study of HPV and oropharyngeal SCC, no evidence was found for any such synergy [17].

HPV-16 has been shown to be causally associated primarily with HPV-cytopositive SCC of the palatal tonsils [14,26,32,37] in subjects who typically are younger, are more engaged with high-risk sexual behaviour (numerous life-time sexual partners and practising oro-genital sex), have higher HPV-16 serum antibody titers, use less tobacco and alcohol, and have a better rate of survival than those subjects with HPV-cytonegative oropharyngeal SCC [9,11-14,33,38].

In these persons with HPV-cytopositive oropharyngeal SCC, the tumour cells have a distinct molecular profile [35]. The cells express transcriptionally active mRNA, frequently show viral integration, high viral load (> 1 copy per cell), functional overexpression of p16^INK4A^, unmutated p53 gene, and decreased levels of pRb; and LoH at chromosomal loci 3p, 9p and 17p is uncommon [14,24,26,35-37,39-41].

In contrast to cells of HPV-cytopositive SCC of the oropharynx as described above, the cells of HPV-cytopositive oral SCC are typically characterised by low viral load, and by infrequent viral integration and by expression of transcriptionally active E6/E7 mRNA [40,42]. A low-copy number (< 1 copy per cell) or absence of transcriptionally active E6/E7 mRNA is indicative of limited biological significance in the oncogenic process [23,35], and of a nonclonal association between the epithelial neoplastic proliferation and the HPV infection [43].

However, it is possible that in some cases of HPV-cytopositive oral SCC that do not express E6/E7 mRNA, the virus has participated in the initial stages of transformation but phased out during later stages [43]; or that HPV super-infection of initially transformed oral keratinocytes may have promoted, in an additive or synergistic manner, the progression of transformation [26].

One must not overlook the fact that not all oral and oropharyngeal SCC are either HPV or tobacco/alcohol related. Some are idiopathic but the proportion of idiopathic to HPV and to tobacco-alcohol induced neoplasms remains undetermined.

## Prophylaxis

In view of the fact that HPV infection is most frequently sexually acquired and that HPV infection is implicated in the aetiology of oropharyngeal SCC, and to a lesser degree in the aetiology of oral SCC, anything that can be done to discourage early sexual activity and to encourage safe sexual practices may reduce the frequency of SCC in anogenital, oral and oropharyngeal sites.

In addition to the encouragement of responsible sexual behaviour, the introduction of HPV vaccination as a public health measure against anogenital HPV infection, will most probably also have a favourable impact on the frequency of HPV-mediated oral and oropharyngeal SCC. The current quadrivalent vaccine against HPV types 6, 11, 16, and 18 consists of L1 protein of HPV which generates a high level of HPV genotype-specific neutralising antibodies [44,45]. The vaccine induces not only a vigorous humoral immune response but also a B cell immune memory response that persists for about 5 years [46].

The quadrivalent vaccine is highly effective (98%) in preventing HPV-16 or HPV-18- related high-grade cervical intraepithelial neoplasia in a population of women aged 15 to 26 who had not been previously exposed to either HPV-16 or HPV-18; but, the vaccine is much less effective in women who have previously been exposed to these HPV types [47]. It is clear, therefore, that vaccination before the onset of sexual activity, which is certainly the primary route of transmission, seems to give the best preventive benefits [48,49].

Genital HPV infection in men appears to be as common as it is in women, is also positively related to a history of sexual activity, but is generally asymptomatic and is therefore an important occult reservoir of the virus, contributing significantly to cervical disease in women. HPV-16 is associated with both penile carcinoma and male oral and oropharyngeal SCC. The conclusion must be that young men before starting sexual activity might also be protected from HPV infection and subsequent oral and oropharyngeal SCC by timeous prophylactic HPV vaccination; and moreover, their sexual partners can also benefit from this preventive measure [48,50].

## Conclusion

• Oropharyngeal SCC to a higher degree, and to a lesser degree oral SCC, are associated with HPV infection.

• Oral and oropharyngeal HPV infection and HPV-related oral and oropharyngeal SCC occur more frequently in persons who have had a number of sexual partners and in those who have practised oral sex.

• Social mouth-to-mouth contact, autoinoculation and vertical birth-transmission are less frequent, but still important routes of transmission of HPV infection.

• The importance of latent HPV infection in the oral and oropharyngeal mucosa as a reservoir of the virus, is undetermined.

• Reliable markers for progression of high-risk HPV-infected epithelium to malignancy are not yet available.

• It is unknown whether co-infection by more than one HPV genotype increases the risk of malignancy, and in the event that it does, whether that malignancy will be more aggressive than that following infection with a single HPV type.

• A number of factors that may well prove to be important in HPV-induced carcinogenesis still remain uncertain:

• the role of immunity;

• variations in genetic profiles of host and virus;

• the specific nature of, and the sequence of the cytogenetic alterations;

• the influences inherent in specific anatomical sites on carcinogenesis.

## Competing interests

The authors declare that they have no competing interests.

## Authors' contributions

LF and RAGK contributed to the literature review. LF, JL and NHW contributed to the conception of the article. LF, JL, NHW and RAG contributed to the manuscript preparation. Each author reviewed the paper for content and contributed to the manuscript. All authors read and approved the final manuscript.
